# Soil texture and microorganisms dominantly determine the subsoil carbonate content in the permafrost-affected area of the Tibetan Plateau

**DOI:** 10.3389/fmicb.2023.1125832

**Published:** 2023-03-21

**Authors:** Ming Shao, Shengyin Zhang, Yu Pei, Sen Song, Tianzhu Lei, Hanbo Yun

**Affiliations:** ^1^Northwest Institute of Eco-Environment and Resources, Chinese Academy of Sciences (CAS), Lanzhou, China; ^2^University of Chinese Academy of Sciences, Beijing, China; ^3^State Key Laboratory of Frozen Soil Engineering, BeiLu’He Station, Northwest Institute of Eco-Environment and Resources, Chinese Academy of Sciences (CAS), Lanzhou, China; ^4^Department of Geosciences and Natural Resource Management, Center for Permafrost (CENPERM), University of Copenhagen, Copenhagen, Denmark; ^5^Department of Earth, Atmospheric and Planetary Sciences, Purdue University, West Lafayette, IN, United States

**Keywords:** soil carbon dynamic, soil texture, microorganisms, pedogenic carbonate minerals, alkaline permafrost regions, Tibetan Plateau

## Abstract

Under climate warming conditions, storage and conversion of soil inorganic carbon (*SIC*) play an important role in regulating soil carbon (C) dynamics and atmospheric CO_2_ content in arid and semi-arid areas. Carbonate formation in alkaline soil can fix a large amount of C in the form of inorganic C, resulting in soil C sink and potentially slowing global warming trends. Therefore, understanding the driving factors affecting carbonate mineral formation can help better predict future climate change. Till date, most studies have focused on abiotic drivers (climate and soil), whereas a few examined the effects of biotic drivers on carbonate formation and *SIC* stock. In this study, *SIC*, calcite content, and soil microbial communities were analyzed in three soil layers (0–5 cm, 20–30 cm, and 50–60 cm) on the Beiluhe Basin of Tibetan Plateau. Results revealed that in arid and semi-arid areas, *SIC* and soil calcite content did not exhibit significant differences among the three soil layers; however, the main factors affecting the calcite content in different soil layers are different. In the topsoil (0–5 cm), the most important predictor of calcite content was soil water content. In the subsoil layers 20–30 cm and 50–60 cm, the ratio of bacterial biomass to fungal biomass (B/F) and soil silt content, respectively, had larger contributions to the variation of calcite content than the other factors. Plagioclase provided a site for microbial colonization, whereas Ca^2+^ contributed in bacteria-mediated calcite formation. This study aims to highlight the importance of soil microorganisms in managing soil calcite content and reveals preliminary results on bacteria-mediated conversion of organic to inorganic C.

## 1. Introduction

Global warming, caused by the atmospheric increase in CO_2_ and other greenhouse gases, is closely linked to the carbon (C) cycle among different C pools (Cox et al., 2000; Thomas et al., 2004; Crowther et al., 2016). The soil C pool, as an important source–sink medium in the C cycle of the terrestrial ecosystem, and the C emission and fixation of soil, directly influences the C cycle of the ecosystem (Raich and Schlesinger, 1992; Davidson and Janssens, 2006; Batjes, 2014). Moreover, the soil C pool is twice the atmospheric C pool and 2-3 times the vegetation C pool (Kerang et al., 2003; Davidson and Janssens, 2006). Due to its large size, slight changes in the balance between inputs to and outputs from the soil C pool would have a significant impact on atmospheric CO_2_ (Schlesinger and Andrews, 2000; Liang et al., 2017; Crowther et al., 2019). Therefore, understanding the soil C dynamics under climate warming can improve confidence in global warming model predictions.

The soil C pool, composed of soil organic carbon (SOC) and soil inorganic carbon (*SIC*) pools, has great potential for carbon sequestration (Tan et al., 2014; Zhang et al., 2015). Most previous studies on alleviation of elevated atmospheric CO_2_ levels have concentrated on the SOC pool because it responds quickly to global climate change, such as warming and nitrogen (N) deposition, and it is strongly linked with various ecosystem functions (Jobbagy and Jackson, 2000; Yang et al., 2012; Pan et al., 2022; Mayer et al., 2023). On the other hand, compared to the relatively short turnover time of SOC, *SIC* has a long resident time in the soil C pool and a long renewal cycle, which is considered to be slow in response to global changes and vegetation succession (Monger et al., 2015; Zang et al., 2018). Thus, little attention has been paid to *SIC* pool dynamics. Nonetheless, it has been reported that alkaline soils in arid and semi-arid regions can absorb CO_2_ from the atmosphere and lead to *SIC* sink, which may affect the soil C cycle (Licht et al., 2022; Vinkovic et al., 2022).

In arid and semi-arid areas, the *SIC* (mainly carbonate) pool is 2-10 times larger than SOC pool (Yang et al., 2010; Wang et al., 2015; Zhang et al., 2015; Guo et al., 2016), and its content and distribution are affected by soil moisture, temperature, depth, salinity, pH, soil type, and parent rock (Raza et al., 2020; Song et al., 2021; Naorem et al., 2022). Especially, in desert ecosystems with sparse vegetation, the increase in soil pH and salinity is conducive to carbonate mineral formation (Gong et al., 2016). Contrary to abiotic factors, the role of microorganisms has been overlooked because the soil microbial activity is poor in desert ecosystems, which have sparse vegetation (Ferdush and Paul, 2021). However, it has also been reported that soil microorganisms can induce carbonate mineral formation and increase the storage of inorganic C pools in arid and semi-arid areas (Pere and Ramn, 2008; Zhao et al., 2020; Pan et al., 2022). Thus, it is important to accurately predict the dynamic change in soil C in a desert ecosystem to determine whether the microbial presence affects carbonate mineral formation.

The Tibetan Plateau, half of which is located in a drought-arid climate, contains 15.2 Pg inorganic C in the top 3 m of grassland (Yang et al., 2010), which is equivalent to its organic C content (15.3 Pg) in the top 3 m of permafrost (Ding et al., 2016). In deserts, the *SIC* stock is much larger than the SOC stock (Mi et al., 2008). In the past several decades, the plateau has experienced significant warming (Qin et al., 2021). This continuous warming has resulted in a significant increase in permafrost thawing rate and microbial activity (Jansson and Tas, 2014; Wu et al., 2015), which can likely induce changes in soil carbonate content and *SIC* stock through biogeochemical processes (Raza et al., 2020; Pan et al., 2022). Hence, an alluvial fan distributed in desert and steppe ecosystems was selected to explore the influence of soil microorganisms on carbonate mineral formation in permafrost regions via phospholipid fatty acid (PLFA) analysis. The hypothesized that has been considered in this work is that microbes have a greater influence on the carbonate content in the topsoil than in the subsoil.

## 2. Materials and methods

### 2.1. Study area

The research area is situated in the permafrost zone of the Beiluhe Basin on the central Tibetan Plateau. According to meteorological data, the Beiluhe Basin has an arid environment with an average yearly temperature in the range of −5.4°C to −3.6°C and mean annual precipitation of 369.8 mm, which is much less than the region’s mean annual evaporation of 1317 mm (Peddle and Franklin, 1993; Ni et al., 2021). *Stipa purpurea* and *Carex rigescens* dominate the alpine steppe site (Zhang et al., 2013). There is no flora growing on the alpine desert’s surface. The area has continuous permafrost, with a maximum depth of 3 m and an average yearly ground temperature of −0.9°C (Niu et al., 2005; Yu et al., 2015). During the thawing season, the active layer arises. Human intervention in this region is minimal, and the region is mostly unaltered.

### 2.2. Sampling and preparation of soils

**FIGURE 1:**
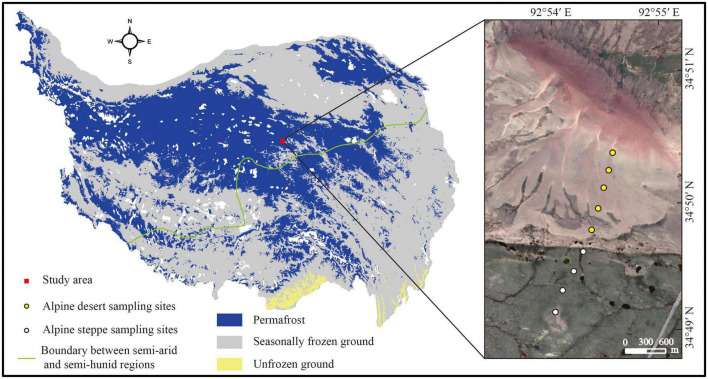
Sampling sites and distribution of permafrost on the Tibetan Plateau. The boundary between the semi-arid and semi-humid regions was modified according to Zhang et al. (2021). The map was created by the authors using ArcMap 10.4 (Environmental Systems Research Institute, Inc., Redlands, CA, USA) (Zou et al., 2017).

At the Beiluhe Long-term Permafrost Research Station (34°50′57.42″N, 92°54′23.96″E, 4,694 m), nine locations with two types of ecosystems were 300–400 m apart and the collection of 27 soil samples occurred in mid-September 2021. The soil from each sampling site was collected in three layers: 0–5 cm, 20–30 cm, and 50–60 cm (Chen et al., 2021). Five sampling sites were chosen from the alpine desert and four were selected from the alpine steppe (Figure 1). Three soil replicates from each sampling site were merged into a single replicating sample using a soil auger with a diameter of 5 cm. Each collected soil sample was sieved (with a 2 mm mesh) to remove big particles and surface vegetation before being split into two subsamples. Fresh samples were used to determine the soil water content (SWC). The remainder of the subsample was air-dried to determine the soil’s physical and chemical characteristics, namely pH, SOC content, soil texture, and mineral composition. The first subsample was kept at −20°C for PLFA analysis (Shao et al., 2022).

### 2.3. Soil property analyses

Samples were dried for 24 h at 105°C in an oven before being given time to absorb as much moisture as they could. The SWC of each soil sample was calculated by gravimetric analysis. A 1:2.5 (w/v) mixture of soil and deionized water was used to measure the pH of the soil using an electrode from a pH meter (PB-10, Sartorius, Germany) (Liu et al., 2020). In a 1:5 (w/v) mixture of soil and deionized water, the electrical conductivity (EC) of the soil was measured using a DDS-307A conductivity meter (Precision and Scientific Corp., Shanghai, China). A CS-902G analyzer was used to find the SOC content after the samples were acidified with 7% HCl to get rid of carbonates and rinsed with deionized water till neutrality (Zhang et al., 2022). The total carbon content (TC) in the soil was directly measured using the CS-902G analyzer, and *SIC* was calculated using the following formula: *SIC* = TC – SOC. The total nitrogen (TN) content in the soil samples were determined using a Kjeldahl nitrogen meter (SKD-200, Shanghai Peiou Analytical Instruments Corp., China), and C/N was calculated. Moreover, the chemical index of alteration (CIA) of all the samples were calculated using the formula: CIA = [Al_2_O_3/_(Al_2_O_3_ + CaO + K_2_O + Na_2_O)] × 100 (Parker, 1970). The supernatant of soil samples after pressurized acid digestion and the deionized water treatment (v/v = 1:5), were measured separately by inductively coupled plasma-optical emission spectroscopy (ICP-OES, Optima800, PerkinElmer, USA) to determine major element concentrations, i.e., Ca, Mg, Fe, Al, K, Na, Ti, and P (Yang et al., 2016).The Cl^–^, NO^–^ 3, and SO2^–^ 4 contents were determined by ion chromatography (ECO IC, Metrohm, Swiss), and the CO2^–^ 3 and HCO^–^ 3 contents were determined according to a Chinese industrial standard (DZ/T 0064. 49-93).

After removing organic matter and carbonates with hydrogen peroxide and HCl, respectively, the soil texture (clay/silt/fine sand/coarse sand%) was categorized in accordance with the international system using a particle size analyzer (Malvern Masterizer 2000, UK) in a measuring range of 0.02–2,000 μm. Mineral composition was evaluated using CuK radiation at 40 kV and 40 mA while operating at divergence, scattering, and reception slits of 1° and 0.15 mm, respectively. The scanning angle spanned from 2° to 52°, the step interval was 0.02°, and the pace was 4°(2)/min. MDI Jade 5 software was used to calculate the mineral contents and percentages of the samples. The Chinese industrial standard SY/T 5163-2010 was used to calculate the mineral content (Sun et al., 2020).

### 2.4. Carbon isotope analysis

The δ^13^C analysis of carbonates was performed using a gas stable isotope mass spectrometer (Thermo Scientific Delta V – GasBench II). Before measurement, plant residues and microbiological shells were picked out under a magnifying glass. After that, each sample was sieved less than 200 mesh. The treated sediment samples were loaded into glass vials and placed in an aluminum heating block of GasBench II. Then the samples (∼0.1 g) were purged with helium for 7 min followed by a treatment with supersaturated phosphoric acid (98%) for 2 h; the reaction temperature was 72°C. Ultimately the CO_2_ generated by the reaction was carried into the Delta V for detection using helium as the carrier gas. NBS-18 was used as the international reference material, while GBW04405 was used as the national reference material. The δ values are reported in per mil relative to the Vienna Pee Dee Belemnite, with an error less than 0.1‰.

### 2.5. PLFA extraction and analyses

We estimated microbial abundances and community composition by PLFAs. PLFAs were isolated from lyophilized soil biomass using the single-phase Bligh and Dyer method (Qin et al., 2021). The combined soil sample (5 g) was extracted twice with a single-phase mixture of chloroform, methanol, and phosphate buffer (1:2:0.8, v/v/v; pH = 7.4), and the extracted sample was vigorously shaken each time for at least 2 h. Water and chloroform in equal parts were added to the solution to divide it into two phases. By sequentially eluting the organic phase with chloroform, acetone, and methanol, the neutral lipids, glycolipids, and phospholipids were extracted from the organic phase on a silica acid column. The phospholipids were moderately alkaline methanolyzed to create fatty acid methyl esters, and the PLFA samples were then kept at −20°C until analysis. The amount of n-tetracosane-D50 (C_24_D_50_), which was added as an internal standard, was the same in each test sample. The isolated PLFAs were then identified and quantified using the MIDI Sherlock Microbial Identification System (Agilent 7890A-5977N) (MIDI Inc., Newark, DE, USA). Bacteria (i14:0, a15:0, i16:0, 16:17c, a17:0, cy17:0, i17:0, 18:17, and cy19:0) and fungi (18:26 and 18:29c) were quantified using particular biomarkers, and the microbial community composition was analyzed using the ratio B/F (Qin et al., 2021). G^+^ PLFAs included i14:0, i15:0, a15:0, i16:0, i17:0 and a17:0; and G^–^ PLFAs included 16:1ω7c, cy17:0 and cy19:0 (Bai et al., 2017). Moreover, the ratio of monounsaturated to saturated fatty acids (MUFA/SFA) was used as a stress indicator to indicate microbial response to environmental stresses (Pivnickova et al., 2010; Sun et al., 2018).

### 2.6. Statistical analyses

The Kolmogorov–Smirnov test and Levene’s test were used to check the normality and homogeneity of the variance of data before conducting statistical analyses. All components were log-transformed because they did not follow a normal distribution, with the exception of pH, SWC, EC, TN, C/N, CIA, MUFA/SFA, coarse sand, and fine sand. To consider multicollinearity among variables before multiple linear regression, the variance inflation factor (VIF) was calculated; acceptable collinearity is defined as a VIF of less than 2 (Craney and Surles, 2002; Qin et al., 2019). SPSS 26.0.0.0 was used for the statistical studies mentioned above at a significance threshold of 0.05.

The following three steps were involved in the statistical analysis after that. First, we looked at significant variations in soil physicochemical parameters and PLFA values between the three depths and two ecosystems using mixed-effects models (R package: nlme). Second, the Pearson correlation was used to examine the relationship between soil environmental properties. Third, linear regression models were used to determine the relationships between calcite content and *SIC* and variables with significant differences in soil depth.

Also, multiple linear regression models were employed to examine soil calcite content and *SIC* with all the variables by using maximum likelihood estimation, which included the variables significantly affecting calcite content and *SIC* in pairwise analyses. The absolute values of the standardized regression coefficients of the explanatory variables accounting for the percentage of the sum of all standardized regression coefficients were used to calculate the relative effect of all parameter estimates to assess the relative significance of the drivers in predicting calcite content and *SIC*. The contribution of various types of predictor factors to a dependent variable was comparable to the simultaneous testing of each predictor variable in the model. Then, to quantify their relative contribution to calcite content and *SIC* in different soil depths across all sites, 21 predictor variables were categorized into four groups, namely edaphic factors (pH, SWC, EC, SOC, TN, C/N, and CIA), soil texture (coarse sand, fine sand, silt, and clay), soil minerals (the contents of quartz, k-feldspar, plagioclase, and clay mineral), and microbial factors (B/F, G^+^/G^–^, MUFA/SFA, microbial biomass, bacterial biomass, and fungal biomass).

## 3. Results

### 3.1. Characteristics of soil properties and microbial factors in different depths and ecosystems

Based on the results of the mixed-effects models, *SIC* and soil calcite content did not exhibit significant differences between the two vegetations (Supplementary Table 1). Soil pH, quartz content, and coarse sand content in the alpine desert were significantly higher than those in the alpine steppe (all *p* < 0.05, Supplementary Table 1), whereas SWC, EC, SOC content, plagioclase content, fine sand content, microbial biomass, bacterial biomass, and fungal biomass showed the opposite results (all *p* < 0.05, Supplementary Tables 1, 2).

Four kinds of soil texture were identified: clay (< 2 μm), silt (2–20 μm), fine sand (20–200 μm), and coarse sand (200–2,000 μm). In all samples, clay made up 0.1–11.8%, silt made up 2.3-17.7%, fine sand made up 44.3-87.7%, and coarse sand made up 1.5–43.4% (Supplementary Table 2). Analysis of the soil’s mineralogy revealed that all samples had similar mineralogical makeup (Supplementary Figure 1). All samples contained quartz, which was predominant (55.5–91.3%), along with calcite (3.5–20.4%), plagioclase (2.4–15.2%), K-feldspar (0.0–10.5%), and clay minerals (0.0–12.6%) (Supplementary Table 1). The results of the soil particle size analysis were consistent with the findings that the clay mineral concentrations of 22 samples were below the instrument’s detection limit (Supplementary Table 2).

**FIGURE 2:**
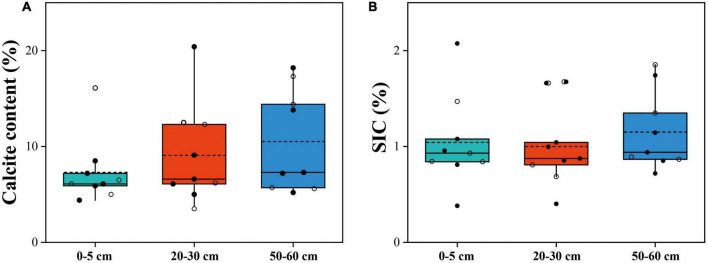
Calcite content **(A)** and *SIC* content **(B)** in soil depths of 0–5 cm, 20–30 cm, and 50–60 cm. The horizontal solid and hollow lines inside each box represent the medians and mean values, respectively. The solid dots represent alpine desert samples, and the hollow dots represent alpine steppe samples.

The SWC of the alpine desert ranged from 3.5 to 23.4%, while that of the alpine steppe ranged from 6.7 to 29.4%. All 27 soil samples were alkaline in nature, with pH values of 8.3–9.0. The SOC content varied from 0.4 to 8.3‰ in the alpine desert and from 1.4 to 6.8‰ in the alpine steppe (Table 1 and Supplementary Table 1). However, there were no significant differences in soil properties (including calcite content and *SIC*) among the three layers, except for the MUFA/SFA value in soil (*p* = 0.035) (Figure 2). The mean MUFA/SFA values for soil layers 0–5 cm, 20–30 cm, and 50–60 cm were 0.50, 0.33, and 0.20, respectively (Table 1 and Supplementary Table 1). This indicated that the available carbon resources in soil decreased significantly with the increasing soil depth. Moreover, the mean B/F value in the 50–60 cm soil layer (mean = 9.98, Table 1) was significantly higher than that in the 0–5 cm soil layer (mean = 4.64, Table 1; *p* = 0.045). With increasing in soil depth, the microbial community structure changed significantly, and the bacterial biomass increased significantly compared with that of fungi (Kieft et al., 1997).

**TABLE 1:** Descriptive statistics of B/F and MUFA/SFA at different depths.

		Bacterial biomass/ Fungal biomass (B/F)	Monounsaturated fatty acid/ Saturated fatty acid (MUFA/SFA)
0–5 cm	Min	2.83	0.16
	Mean	4.64	0.50
	Max	7.49	0.80
20–30 cm	Min	2.72	0.06
	Mean	5.40	0.33
	Max	8.80	0.71
50–60 cm	Min	3.92	0.04
	Mean	9.98	0.20
	Max	25.66	0.45

### 3.2. Associations of soil carbonate and *SIC* contents with soil properties and microbial factors

**FIGURE 3:**
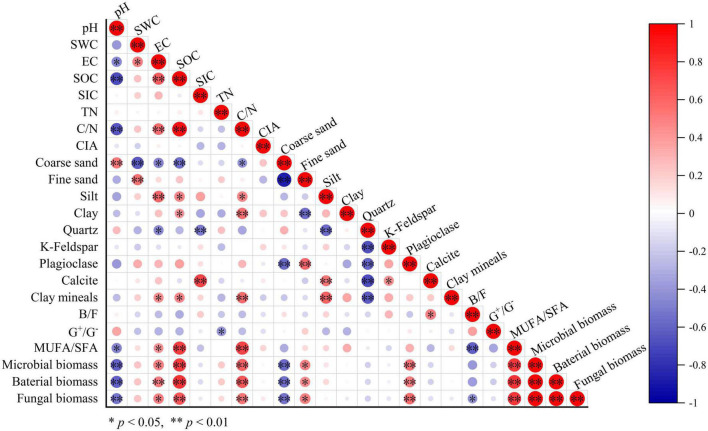
Pearson correlation analysis results of soil variables in all samples.

We established a relationship between carbonate content and soil properties in all samples. The calcite content in soil was significantly associated with the quartz, k-feldspar, and silt contents (all *p* < 0.05, Figure 3). Particularly, soil calcite content was positively correlated with *SIC* and the B/F values (*p* = 0.011). Moreover, there was also a significant correlation between *SIC* and quartz content in soil (*p* = 0.007, Figure 3).

**FIGURE 4:**
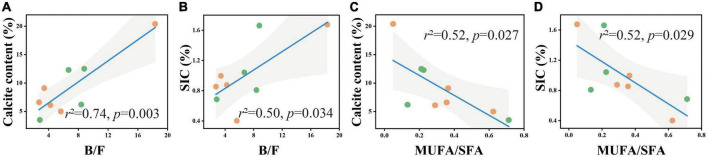
B/F and MUFA/SFA in relation to soil calcite content **(A,B)** and *SIC* content **(C,D)** in the soil depth of 20–30 cm. The solid lines are fitted by ordinary least-squares regressions, and the shadow areas correspond to 95% confidence intervals. The orange dots represent alpine desert samples, and the green dots represent alpine steppe samples.

**FIGURE 5:**
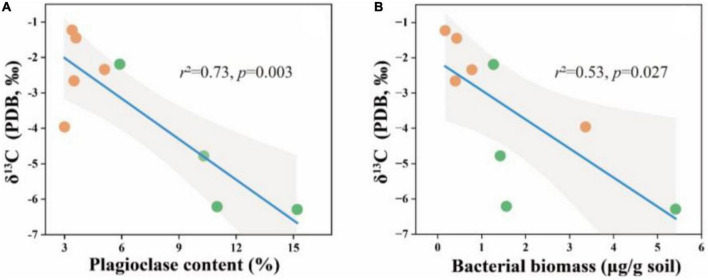
δ^13^C in relation to soil plagioclase content **(A)** and bacterial biomass **(B)** in the soil depth of 20–30 cm. The solid lines are fitted by ordinary least-squares regressions, and the shadow areas correspond to 95% confidence intervals. The orange dots represent alpine desert samples and the green dots represent alpine grassland samples.

To better understand the relationship between carbonate content and microbial community structure, linear regression models were used to characterize the correlation between the two for each soil layer. In the 0–5 cm and 50–60 cm soil layers, calcite content and *SIC* had no significant correlation with B/F and MUFA/SFA. In the 20–30 cm soil layer, calcite content and *SIC* had a significant positive correlation with B/F (Figures 5A, B) and a significant negative correlation with MUFA/SFA (Figures 4C, D).

Furthermore, we tested the carbon isotopes of soil carbonate minerals to confirm the influence of microorganisms on soil carbonate. The results showed that the δ^13^C values in soil carbonate were significantly associated with plagioclase content and bacterial biomass (Figures 5A, B). Lower δ^13^C values of soil carbonate were reported, the increasing plagioclase mineral content and bacterial biomass in the soil.

### 3.3. Determinants of soil carbonate content and *SIC* at different depths

**FIGURE 6:**
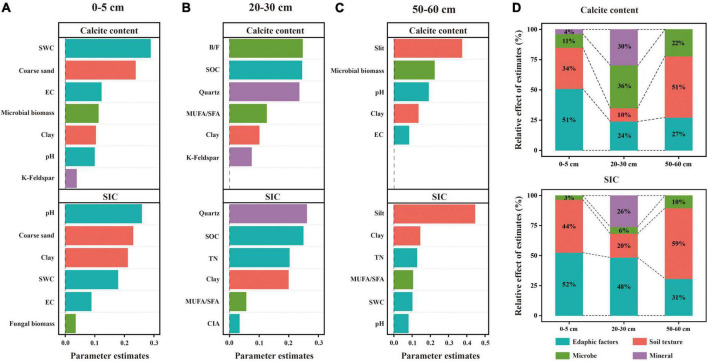
Relative effects of multiple drivers of soil calcite and *SIC* contents in the soil depths of 0–5 cm **(A)**, 20-30 cm **(B)**, and 50–60 cm **(C)**. The relative effects of four factors (edaphic factors, soil texture, microbe and mineral) on calcite and SIC contents were calculated **(D)**. Edaphic variables include pH, SWC, EC, SOC, SON, and CIA; soil texture includes coarse sand, silt, and clay; microbial variables include B/F, MUFA/SFA, microbial biomass, and fungal biomass; and soil minerals include quartz and k-feldspar.

The dominant factors affecting the soil carbonate and *SIC* contents were different among the three soil layers. Specifically, for the 20–30 cm soil layer, the linear model revealed that microbial and mineral factors largely contributed to the variations in the calcite content, followed by edaphic factors, and soil texture contributed the least (Figure 6D). Among these factors, B/F, SOC, and quartz content exhibited larger effects on the calcite content compared with other predictor factors (Figure 6B). For the 0–5 cm soil layer, the linear model showed that edaphic factors largely explained the variations in the content of calcite, followed by soil texture and microbial factors and lastly soil mineral (Figure 6D). Among these factors, SWC and the coarse sand content had larger contributions to the variation in calcite content than others (Figure 6A). For the 50–60 cm soil layer, the linear model showed that soil texture largely explained the variations in the calcite content, followed by edaphic factors, and lastly microbial factors (Figure 6D). Among these factors, silt content had larger contributions to the variation of calcite content than others (Figure 6C).

Similarly, linear models showed that edaphic factors and soil texture largely explained the variations in *SIC* for the 0–5 cm and 50–60 cm soil layers, respectively (Figure 6E). The difference, however, was that pH exhibited larger effects on *SIC* compared with other predictor factors in the 0–5 cm soil layer (Figure 6A). Moreover, edaphic factors largely explained the variations in *SIC* in the 20–30 cm soil layer, followed by soil mineral and soil texture and lastly microbial factors (Figure 6D). With increasing soil depth, the influence of edaphic factors on *SIC* decreased while the influence of microorganisms on *SIC* increased.

## 4. Discussion

### 4.1. Effect of soil bacteria community on pedogenic carbonate formation

Due to considerably stable characteristics and the long turnover time, *SIC* stock is traditionally considered to be dominated by abiotic factors including soil moisture, soil pH, CO_2_ partial pressure, and Ca^2+^ concentrations (Yang et al., 2012; Rey, 2015; Yang and Yang, 2020). However, our results revealed that the influence of microbial factors on the variation in *SIC* gradually increased with soil depth, and B/F was the strongest predictor for the change in calcite content among microbial factors in the 20–30 cm soil layer (Figure 6B). The significant positive correlation between calcite content and B/F suggested that the higher the bacterial biomass in soil was beneficial to the formation of calcite minerals compared with the fungal biomass (Figures 3, 4A, B). Furthermore, the significant negative correlation between bacterial biomass and carbonate carbon isotopes not only proved that carbonate minerals were mainly secondary in the 20–30 cm soil layer of the study area, but also proved the influence of bacteria on the carbonate mineral formation (Figure 5B).

**FIGURE 7:**
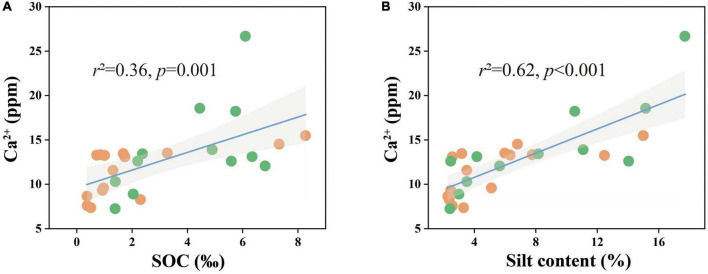
Ca^2+^ concentrations in relation to **(A)** SOC content and **(B)** soil silt content in all samples. The solid lines are fitted by ordinary least-squares regressions, and the shadow areas correspond to 95% confidence intervals. The orange dots represent alpine desert samples and the green dots represent alpine steppe samples.

Combined with the significant negative correlation between calcite content and MUFA/SFA (Figures 4C, D), we suggested that bacteria-mediated calcite formation is related to resource competition against fungi. In arid and semi-arid regions, where SOC is very low, competition for resources affects the fungi and bacteria biomass (Figure 3; Bahram et al., 2018). Also, high free Ca^2+^ concentrations in alkaline soil contribute to SOC stabilization through the form of cation bridging (Rowley et al., 2018). This is consistent with our result that SOC has a significant positive correlation with the Ca^2+^ concentration (Figure 7A). This form of cation bridging by Ca^2+^ (Ca-OC) has been highlighted as an important component of SOC stabilization by many authors and is well-documented (Oades, 1988; Clough and Skjemstad, 2000; Edwards and Bremner, 2010). To survive, bacteria have to decompose Ca-OC because of the broader activities of fungi than bacteria in using complex carbon substrates (Bahram et al., 2018). Thus, bacteria would mediate the calcite mineral formation to obtain OC bound to Ca^2+^ in two types: biologically controlled and induced mineralization (Dong et al., 2022). Biologically controlled mineralization is the active absorption of calcium ions by microbial cells and the formation of carbonate precipitation by regulating the saturation conditions of cells (Bazylinski and Frankel, 2004; Benzerara et al., 2011). Biological induced mineralization is a passive process in which microorganisms alter the pH through metabolic activities and bind large amounts of calcium ions to their cell surface and extracellular polymers, making their microenvironment conducive to carbonate mineral formation (Muynck et al., 2010; Phillips et al., 2013).

It was found that as the proportion of available organic carbon decreases, bacteria can take advantage of the more stable organic matter that binds to calcium ions through mediating CaCO_3_ formation in alkaline soil environments. However, this process can be heavily influenced by external factors, including edaphic factors and soil texture, which results in the discovery only in the 20–30 cm soil layer of this study area. Moreover, SOC can also be stabilized through either inner- or outer-sphere interaction with minerals (Sposito, 1989; Sutton et al., 2010). Thus, plagioclase can provide a site for microbial colonization, leading to a significant positive correlation between plagioclase content and microbial biomass in soil with little clay mineral content (Figure 3). At the same time, the negative correlation between plagioclase content and carbonate carbon isotope in the 20–30 cm soil layer indicated that plagioclase provided the Ca^2+^ for bacteria-mediated calcite formation (Figure 5A).

### 4.2. Effect of soil texture on pedogenic carbonate and *SIC*

Although much attention has been paid to the effect of microorganisms on soil carbonate content in recent years, the effect of soil texture on *SIC* pools has not been studied. However, our results showed that the silt content in the soil had a significant relationship with calcite content and played a dominant role in regulating the calcite content and *SIC* stock in the 50–60 cm soil layer (Figures 3, 7C). Soil texture is fundamentally affected by soil mineral composition and content: the higher the quartz content, the lower the feldspar content, and the more difficult it is to be weathered (Franke, 2009). The quartz content in the soil of the study area was much higher than those of other minerals, resulting in the soil texture being sandy soil and significantly affecting the silt content (Figure 3 and Supplementary Table 2). The silt content was positively correlated with the water-soluble ion content and EC, especially the Ca^2+^ content (Figure 7B and Supplementary Table 2). Thus, the silt content of soil can not only directly affect the calcite content by controlling soil the Ca^2+^ content of soil but also indirectly affect the calcite content through EC affecting microbial activity (Yuan et al., 2007; Prindle et al., 2015; Yan et al., 2015).

Taken together, our results demonstrate that in arid and semi-arid areas, bacteria acquire Ca-OC in the soil environment by mediating calcite mineral formation for survival, leading to the conversion of organic carbon to inorganic carbon. Moreover, the effect of soil texture on soil biogeochemical processes was further amplified with increasing in soil depth.

## 5. Conclusion

This study examined the evidences for the relative contributions of edaphic factors, microorganisms, and soil textures to the variation in calcite content and *SIC* stocks at different soil depths, which has considerable implications for grasping the importance of *SIC* in the C cycle. Our results showed that in arid- and semi-arid areas, *SIC* and soil calcite contents did not exhibit significant differences among the three soil layers. However, the main factors affecting the calcite content in different soil layers (0–5 cm, 20–30 cm, and 50–60 cm) are different, which are edaphic factors, microbial factors, and soil texture, respectively. In the topsoil (0–5 cm), the most important predictor of calcite content was SWC, whereas the most important predictor of *SIC* was soil pH. In the subsoil (20–30 cm and 50–60 cm), the B/F ratio and soil silt content, respectively, had larger contributions to the variation of calcite content than others in the subsoil. Bacteria were the main microorganisms that mediated the formation of secondary carbonate minerals in the 20–30 cm soil layer. With increasing soil depth, the influence of edaphic factors on *SIC* decreased, whereas the influence of microorganisms on *SIC* increased. Moreover, plagioclase not only provided a site for microbial colonization, but also provided Ca^2+^ for bacteria-mediated calcite formation. Therefore, for further development of the model, the effects of soil texture and microorganisms on calcite and *SIC* contents should be incorporated to accurately predict permafrost C dynamics and its associated climate feedback.

## Data availability statement

The raw data supporting the conclusions of this article will be made available by the authors, without undue reservation.

## Author contributions

MS: conceptualization, methodology, software, writing—original draft, data curation, investigation, and experiment. SZ: writing—review and editing, methodology, investigation, and data curation. YP: investigation and writing—review and editing. SS: investigation. TL: resources, instrumentation, and funding acquisition. HY: conceptualization and writing—review and editing. All authors contributed to the article and approved the submitted version.
