# Supplementary material for: Soil texture and microorganisms dominantly determine the subsoil carbonate content in the permafrost-affected area of the Tibetan Plateau
Source: Front Microbiol. 2023 Mar 21;14:1125832. doi: 10.3389/fmicb.2023.1125832 (PMC10070835; doi:10.3389/fmicb.2023.1125832)
Supplement: Supplementary file 1 [file Data_Sheet_1.PDF]

Supplementary Material

1 Supplementary Figures and Tables

1.1 Supplementary Figures

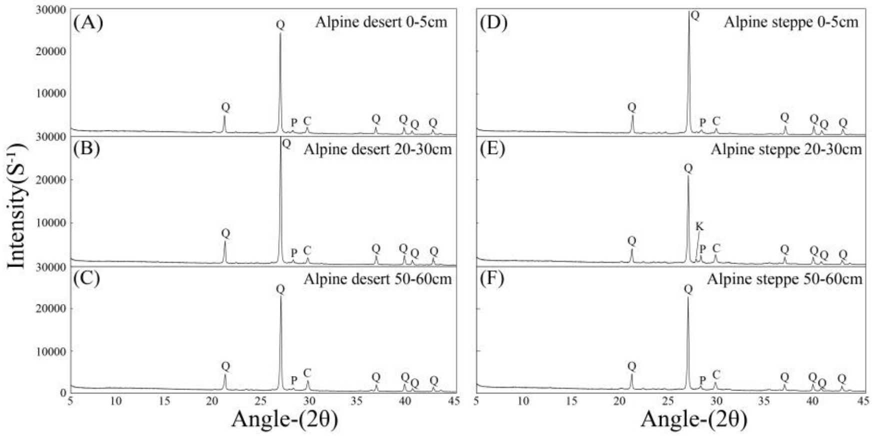

Supplementary Figure 1. X-ray diffraction patterns of the typical samples. The 0−5 cm layer of the alpine desert (A); the 20−30 cm layer of the alpine desert (B); the 50−60 cm layer of the alpine desert (C); the 0−5 cm layer of alpine steppe (D); the 20−30 cm layer of the alpine steppe (E); and the 50−60 cm layer of the alpine steppe (F). Abbreviations for various minerals: Q-, quartz; C-, calcite; P-, plagioclase; K-, potassium feldspar.

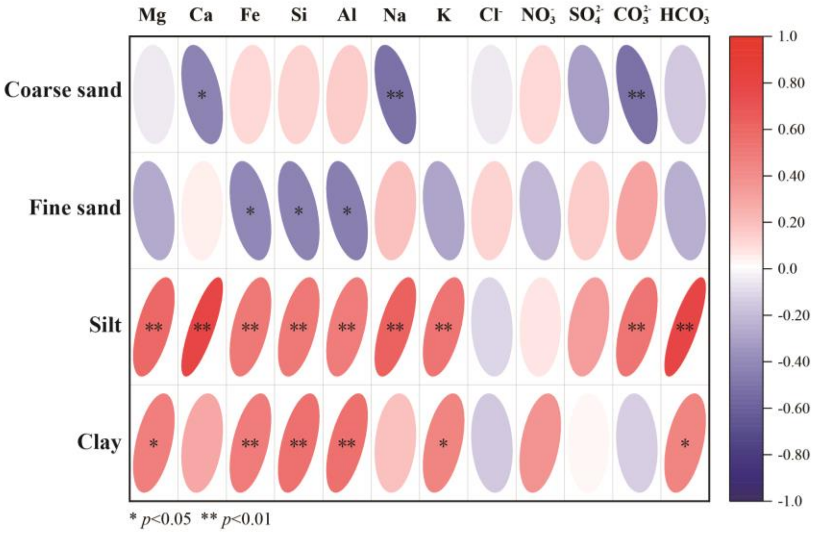

Supplementary Figure 2. Person correlation of soil texture with deionized water extracts cations and anions in all samples.

1.2 Supplementary Tables

Supplementary Table 1. Results of mixed-effects models showing the effects of depth (0-5 cm, 20-30 cm, and 50-60 cm), and ecosystems (alpine desert and alpine steppe), respectively. Asterisks indicate significant influence (** indicates $p < 0.01$; * indicates $p < 0.05$).

Variables	pH	SWC	EC	SOC	SIC	TN	C/N	CIA	Coarse sand	Fine sand	Silt	Clay
Depth	0.765	0.064	0.996	0.841	0.281	2.520	0.664	1.228	0.537	0.111	0.739	0.428
Ecosystem	5.598*	4.233*	0.519	5.103*	0.178	1.400	1.948	0.130	11.524**	9.524**	2.298	2.565
Depth* Ecosystem	0.268	0.174	0.095	0.493	0.105	0.539	0.75	0.010	0.026	0.045	0.165	0.484

Variables	Quartz	K-Feldspar	Plagioclase	Calcite	Clay mineral	B/F	G^+/G^-	MUFA/SFA	Microbial biomass	Bacterial biomass	Fungal biomass	Ca^{2+}
Depth	1.340	2.774	1.026	1.021	0.950	2.448	2.032	6.238*	2.299	2.375	2.302	0.352
Ecosystem	6.534*	2.984	19.943**	0.080	1.369	0.184	0.238	0.265	7.734**	7.164*	5.104*	2.584
Depth* Ecosystem	0.612	0.905	3.702*	0.174	1.080	0.115	0.731	0.224	0.923	0.991	1.039	0.116

SWC: soil water content, EC: electrical conductivity, SOC: soil organic carbon, SIC: soil inorganic carbon, TN: soil organic nitrogen, CIA: chemical index of alteration, B/F: the ratio of bacterial biomass to fungal biomass.

Supplementary Table 2. Descriptive statistics of soil physicochemical properties in different ecosystems.

	pH	SWC/%	EC (μS/cm)	SOC/‰	SIC/%	TN/‰	C/N	CIA/%	Coarse sand/%	Fine sand/%	Silt/%	Clay/%	Quartz/%	K-Feldspar/%	Plagioclase/%	Calcite/%	Clay mineral/%
Min	8.26	3.49	39.20	0.37	0.38	0.30	0.51	13.18	1.50	44.26	2.27	0.12	55.50	0.00	2.40	3.50	0.00

All samples	Mean	8.60	12.16	64.45	3.00	1.06	0.54	5.80	45.20	25.12	66.34	6.65	1.88	81.07	1.82	5.65	8.97	1.79
	Max	9.02	29.39	94.10	8.27	2.07	0.90	18.25	70.33	43.44	87.71	17.70	11.76	91.30	10.50	15.20	20.40	12.60
Alpine desert	Min	8.29	3.49	39.20	0.37	0.38	0.30	0.51	24.99	23.18	44.26	2.27	0.12	70.70	0.00	2.40	4.40	0.00
	Mean	8.69	9.71	61.84	2.11	1.03	0.51	4.62	46.95	32.75	59.05	5.45	2.76	85.09	1.17	3.85	8.73	1.01
	Max	9.02	23.38	89.40	8.27	2.07	0.74	18.25	70.33	43.44	74.24	15.00	11.76	91.30	5.50	5.60	20.40	8.30
Alpine steppe	Min	8.26	6.72	43.80	1.39	0.69	0.33	2.05	13.18	1.50	50.02	2.41	0.17	55.50	0.00	3.90	3.50	0.00
	Mean	8.50	15.23	67.73	4.11	1.10	0.58	7.28	43.01	15.59	75.47	8.16	0.79	76.06	2.64	7.89	9.27	2.78
	Max	8.76	29.39	94.10	6.81	1.85	0.90	12.25	56.17	40.89	87.71	17.70	1.87	88.50	10.50	15.20	17.30	12.60

Supplementary Table 3. Descriptive statistics of microbial properties in different ecosystems.

		B/F	G^+/G^-	MUFA/SFA	Microbial biomass (μg/g soil)	Bacterial biomass (μg/g soil)	Fungal biomass (μg/g soil)
All samples	Min	2.72	0.56	0.04	0.23	0.09	0.01
	Mean	7.02	1.16	0.35	3.30	2.24	0.56
	Max	25.66	2.72	0.80	14.25	9.56	3.01
Alpine desert	Min	2.72	0.74	0.04	0.23	0.09	0.01
	Mean	6.61	1.19	0.32	1.64	1.12	0.25
	Max	18.32	2.72	0.79	5.21	4.20	0.98
Alpine steppe	Min	2.83	0.56	0.04	1.09	0.71	0.04
	Mean	7.51	1.12	0.38	5.23	3.53	0.92
	Max	25.66	1.49	0.80	14.25	9.56	3.01
